# Lost individual income due to severe health events: life-course perspective in the Northern Finland Birth Cohort 1966

**DOI:** 10.1093/eurpub/ckac110

**Published:** 2022-08-30

**Authors:** Ina Rissanen, Iiro Nerg, Leena Ala-Mursula, Marko Korhonen

**Affiliations:** Department of Epidemiology, Julius Center for Health Sciences and Primary Care, University Medical Center Utrecht and Utrecht University, Utrecht, The Netherlands; Center for Life Course Health Research, University of Oulu, Oulu, Finland; Medical Research Center Oulu, Oulu University Hospital, Oulu, Finland; Department of Economics, Oulu Business School, University of Oulu, Oulu, Finland; Center for Life Course Health Research, University of Oulu, Oulu, Finland; Medical Research Center Oulu, Oulu University Hospital, Oulu, Finland; Department of Economics, Oulu Business School, University of Oulu, Oulu, Finland

## Abstract

**Background:**

Severe health events may lead to reduced income among survivors. Importantly, individuals’ risks for both severe health events and for lower income are shaped by early life course. Our aim was to consider early-life factors in determining lost individual income after stroke, heart attack and cancer between ages 18 and 50.

**Methods:**

A population-based Northern Finland Birth Cohort 1966 (*N* = 12 058) was used. Early-life factors were collected since mid-pregnancy until age 16 years and used to match all persons with stroke, heart attack, or cancer (*n* = 995) with four controls. Registered annual individual income development 15 years before and after the event was compared between cases and propensity score matched controls using time-to-event mixed models, stratified for sex.

**Results:**

Compared to controls, a new decreasing income trend emerged among women after stroke (logarithmic income per time −0.54; 95% CI −0.88 to −0.20), whereas men getting stroke showed declining earnings already by the time of the event, further declining after stroke (−1.00, −1.37 to −0.63). Getting heart attack was associated with a new declining trend both in women (−0.68; −1.28 to −0.09) and men (−0.69, −1.05 to −0.32). Income declined also among control men (−0.24, −0.34 to −0.14), who had higher income but were less educated than control women.

**Conclusions:**

Stroke and heart attack but not cancer have exogenous deleterious effects on individual economy, independently of early-life factors. The effects accelerate by time. Negative income trend in control men shows that severe health events do not explain all decrease in income.

## Introduction

Severe health events threaten persons’ lives and cause permanent disabilities, which in turn reduce survivors’ ability to work and earn income, also in long-term.^1–5^ They depress incomes through the loss of employment, but also through reduced income among those who remain employed.^3^ However, social security systems in European countries seem to reduce the individual economic burden of severe health events.^6^

In previous literature, cardiovascular and cerebrovascular diseases as well as cancers have been considered severe health events.^2^ It is thought that a severe health event is an unpredicted, acute, and sudden exogenous shock. However, the unpredictability and acuteness of these diseases has been questioned. The life-course approach of diseases suggests that exposures as early as in prenatal period affect the occurrence of severe health events in adulthood.^7–9^ The early life factors, such as prenatal maternal smoking, birth weight, childhood growth, and nutrition, are socially patterned,^10^ and important mechanisms in generating social inequalities in adult health.^11^ In adulthood, they are also associated with lower individual income^12^ and increased vulnerability to macroeconomic crises.^13^ To date, previous population studies on lost income due to severe health events have failed to consider possible common underlying factors between risk of disease and lower income. To understand the exogenic economic impacts of severe health events, potential underlying life-course factors should be considered.

Our aim was to investigate changes in longitudinal income development due to stroke, heart attack, or cancer, as compared to matched controls in relation to early life factors. Respecting the known differences in the timing and types of various health events during the life course as well as in the division of occupations and levels of income, we expected to find differences in these associations between men and women. A prospective birth cohort of more than 12 000 people with individual-level data linked with national registers is used. We observe accurate administrative data on their individual income during prime working-age. We use the method of propensity score matching to estimate what the levels of income would have been for each person if the severe health event had not occurred. We have full records for each cohort member throughout the sample period.

## Materials and methods

### Study population

The Northern Finland Birth Cohort 1966 (NFBC1966) is a non-selective, prospective, population-based birth cohort followed-up since mid-pregnancy.^14^^,^^15^ The cohort is based on 12 058 live-born children whose expected birth date was in 1966, representing 96% of children born in Finnish Provinces of Lapland and Oulu in 1966. In this study, we used survey and clinical data collected during pregnancy and at birth and survey data at age 14 years, individually linked with nationally registered data.

From the entire cohort, all those who had had a severe health event, i.e. stroke, heart attack, or cancer, until the end of year 2019 were identified (*n* = 995). Diagnoses were obtained from the National hospital discharge register and National cancer register, from which the subjects with International Classification of Diseases (ICD) codes listed in Supplementary table S1 were identified. The data included the date of the disease onset.

Excluded from the sample were persons whose severe health event occurred before reaching adulthood, i.e. before year 1984 (*n* = 69), and persons without at least one positive measurement of annual income (*n* = 1335). The total sample was 10 761 individuals.

Permission to gather register data was obtained from the Ministry of Social Affairs and Health, and the study was approved by the Ethics Committee of Northern Ostrobothnia Hospital District in Oulu, Finland. Mothers of the NFBC1966 members gave informed verbal consent in the beginning of the NFBC1966 in 1965–66. In the later stages of the study, written informed consent has been obtained from NFBC1966 members participating in the follow-ups. The NFBC1966 data are administered by the NFBC Project Center, and researchers who have been granted access to the data are allowed to handle it in a pseudonymized format.

### Matching for early life factors

Within the NFBC1966, we matched every person with severe health event with four controls based on early life characteristics that are known to associate both with increased risk for severe health event and decreased income in adulthood^7^^,^^16^ (Supplementary figure S1). Sex and birth weight were obtained from cohort member’s birth certificates. Mother’s smoking, mood, and attitude towards economic self-reliance during pregnancy, family’s highest occupational status, living environment, and wealth at the time of birth were obtained from questionnaires during the antenatal period. Smoking, alcohol use, BMI, physical activity, and general health in adolescence were obtained from questionnaires at the age of 14 years. Success as grade point average when leaving basic education at age 16 years and grade in sports were obtained from national secondary school application registers.

### Income and employment data

The register of The Finnish Tax Administration^17^ was used to obtain individual level data on the annual gross income of cohort members, available from 1995 to 2016. The taxable individual income includes wage and salary earnings, self-employment income, public income transfer payments, and social security benefits (usually earnings-based). Capital income is not included in this study. The annual values of past income were discounted to the reference year 2019. We used the consumer price index as a converter.^18^ In the analyses, logarithmic annual income was used.

Information on education, occupation, and socioeconomic status was collected from Statistics Finland (https://www.tilastokeskus.fi/en/luokitukset/ammatti/, https://www.tilastokeskus.fi/en/luokitukset/koulutus/, https://www.tilastokeskus.fi/en/luokitukset/sosioekon_asema/). As baseline characteristics, working status, earning days, education, occupation, and socioeconomic status of persons with severe health events and their matched controls are presented. Based on data of the Finnish Center of Pensions^19^ recording all days with work contract or in self-employment, a person was considered to work at the time of severe health event if at least 15 earning days were registered during the month before the event. A person was considered to work one year after the severe health event if at least 180 salaried or self-employed earning days were recorded during the year after the event.

### Statistical analyses

Of the early-life data used for matching, 3.6% were missing and were imputed. The multiple imputation procedure was conducted ten times with the mice-function from the R package ‘mice’.^20^ Early-life factors were both imputed and used as predictors in the model except for sex and birthweight that had no missing values. Sex and birthweight were used only as predictors in imputation model. Missing data indicator, highest occupation in family, income, or diagnosis of interest were not used as predictors for imputations. The imputation process was iterated 40 times and visually inspected that there were no clear trends in the process.

Nearest neighbour matching with propensity scores and without replacement was used to find controls for each person with severe health event based on early-life characteristics (Supplementary figure S2). Exact match on sex and rounded birthweight was required. We aimed for 4:1 ratio for controls and cases, but this was not obtained for all strata in persons with cancer, so the weights from the matching model were used in the final models. We used the MatchThem-function from the R package ‘MatchThem’.^21^ Missing data indicator was used in the matching model. The matching was done for all severe health events combined as we assumed the effect of health shock on income to be similar in all disease categories (stroke, heart attack, cancer).

Time-to-event mixed models were used to investigate the development of income 15 years before and after severe health event compared to matched controls. Time-to-event is calculated as years to the event, prior years having negative values and years after having positive values. For matched controls, the time of event is set to be the same as the time of event of their matched case. Follow-up ended at the time of death, when a person moved permanently abroad, or 31 December 2016. Due to skewed distribution, income was modelled on logarithmic scale. Individual level random intercepts and slopes were used in the models. Linear and non-linear (quadratic and cubic) models were tested, and the non-linear model with quadratic time-to-event term was best fitted to the data. The equation of the model was:

$${\log(\mathrm{wage})}_{ij}=\;\beta_{0}+\;\beta_{1}\times {\mathrm{DT}}_{i}\times {\mathrm{TTE}}_{ij}+\beta_{3}\times {\mathrm{DT}}_{i}\times {\mathrm{TTE}}_{ij}^{2}+u_{0i}+\;u_{1i}\times {\mathrm{TTE}}_{ij}+\varepsilon_{ij}$$

where ${\mathrm{DT}}_{i}$ is the type of disease for an individual i; i=1, …, n; ${\mathrm{TTE}}_{ij}$ is the time to event in years for an individual i at the time point j; j= -15, -14, …, 14, 15; and $u_{0i}$ = random intercept for an individual i. To fit the models, the lmer-function from R package ‘lme4’ was used.^22^ Mixed models were run separately for men and women.

### Data availability statement

NFBC1966 data are available from the University of Oulu, Infrastructure for Population Studies. Permission to use the data can be applied for research purposes via electronic material request portal at the cohort website (www.oulu.fi/nfbc) or by contacting NFBC project centre (NFBCprojectcenter@oulu.fi). In accordance with the EU general data protection regulation (679/2016) and Finnish Data Protection Act, the use of personal data is based on cohort participant’s written informed consent at his/her latest follow-up study, which may cause limitations to its use.

## Results

### Characteristics of sample

The median individual income of women was lower than that of men in the beginning (€15 704 vs. €19 672, Mood’s median test *P* < 0.001) and at the end of follow up (€28 553 vs. €32 944, *P* < 0.001). However, women had more often high education (52% vs. 35%, chi-square *P* < 0.001), and were less often in manual work (13% vs. 29%, *P* < 0.001) than men.

In total, 995 severe health events (556 in women and 439 in men) occurred within the NFBC1966 between 1984 and 2019 (table 1). The incidence rate was 2.7/1000 person years. Of health events, 221 were strokes, 155 were heart attacks, and 619 were cancers with incidence rates of 0.6, 0.4, and 1.7/1000 person-years, respectively. Women had higher cancer incidence rate (2.3 vs. 1.1/1000 person-years in women and men, respectively) but lower heart attack incidence rate (0.2 vs. 0.6/1000 person-years) than men. The mean age at the time of health shock was 43.0 (SD 8.1) years (Supplementary figure S3).

**Table 1:** Characteristics of sample

	All	Women (*N* = 5273)							Men (*N* = 5488)				
	Total sample (*N* = 10 761)		Unmatched control (*N* = 2513)	Control (*N* = 2204)	Stroke (*N* = 108)	Cancer (*N* = 413)	Heart attack (*N* = 35)	Unmatched control (*N* = 3296)		Control (*N* = 1753)	Stroke (*N* = 113)	Cancer (*N* = 206)	Heart attack (*N* = 120)
**Incidence rate per 1000 person years**													
	–		–	–	0.60	2.28	0.19	–		–	0.60	1.09	0.63
**Age at time of health shock, mean (SD)**													
	43.0 (8.1)		–	–	43.5 (8.1)	42.0 (8.2)	44.5 (8.3)	–		–	42.5 (8.2)	43.7 (8.3)	44.8 (6.3)
**Median income**													
In 1995	17 470		16 330	15 355	15 022	15 324	13 699	20 165		19 006	17 201	18 870	17 773
In 2005	26 436		24 219	23 054	20 351	24 480	20 768	30 578		29 720	27 484	29 495	26 919
In 2016	30 326		29 291	27 841	19 400	28 705	24 890	33 215		33 590	23 584	32 730	26 041
**Working before and after the event, *n* (%)**													
Work-work	6944 (64.5)		1954 (77.8)	1122 (50.9)	43 (39.8)	206 (49.9)	11 (31.4)	2544 (77.2)		892 (50.9)	44 (38.9)	74 (35.9)	54 (45.0)
Work-no	661 (6.1)		119 (4.7)	147 (6.7)	13 (12.0)	43 (10.4)	1 (2.9)	195 (5.9)		93 (5.3)	11 (9.7)	29 (14.1)	10 (8.3)
No-work	344 (3.2)		111 (4.4)	66 (3.0)	1 (0.9)	8 (1.9)	1 (2.9)	98 (3.0)		47 (2.7)	2 (1.8)	7 (3.4)	3 (2.5)
No-no	2812 (26.1)		329 (13.1)	869 (39.4)	51 (47.2)	156 (37.8)	22 (62.9)	459 (13.9)		721 (41.1)	56 (49.6)	96 (46.6)	53 (44.2)
**Stayed in the same occupation after severe health event, *n* (%)**													
	3186 (48.8)		933 (44.6)	499 (62.9)	14 (43.8)	105 (63.6)	4 (44.4)	1183 (44.2)		367 (59.0)	21 (52.5)	34 (57.6)	26 (63.4)
**Percentage of earning days after severe health event, *n* (%)**													
Over 80%	6660 (70.0)		1891 (75.2)	1157 (68.2)	42 (51.2)	201 (62.2)	14 (58.3)	2319 (70.4)		883 (70.0)	39 (44.3)	63 (45.0)	51 (58.0)
Over 50%	964 (10.1)		217 (8.6)	225 (13.3)	9 (11.0)	39 (12.1)	1 (4.2)	318 (9.6)		116 (9.2)	14 (15.9)	15 (10.7)	10 (11.4)
Under 50%	1888 (19.8)		405 (16.1)	314 (18.5)	31 (37.8)	83 (25.7)	9 (37.5)	659 (20.0)		263 (20.8)	35 (39.8)	62 (44.3)	27 (30.7)

Income discounted to year 2019. For cases and matched controls, working before the event was considered if a person worked at least 15 days during the previous month of health event, and working the after the event was considered if a person worked at least 180 days during the year after the health event. For unmatched controls, working before was considered if a person worked at least 180 days in the year 2008, and working afterwards if a person worked at least 180 days in the year 2010. Unmatched controls were considered to have stayed in the same occupation if their occupation was same in 2008 and 2011. Cases and propensity-score matched controls were considered to stay in the same occupation if their occupation was same one year before and two years after the event. Earning days were calculated after the year 2010 for unmatched controls.

SD, standard deviation.

At the time of severe health event, 539 (54.2%) persons were working, [317 (57.0%) women and 222 (50.6%) men] (table 1). One year after the severe health event, 454 (45.6%) persons were working [270 (48.6%) women and 184 (41.9%) men]. Of those who were working at the time of the event, 18.0% of women and 22.5% of men did not work one year afterwards. Socioeconomic characteristics at the time of severe health events are shown in Supplementary table S2.

### Severe health events and income

The individual income development between 1995 and 2016 of matched controls and the rest of the NFBC1966 population, stratified by sex, is shown in Supplementary figure S4. Matched controls did not show different income development to the unmatched population (data not shown).

**Figure 1:**
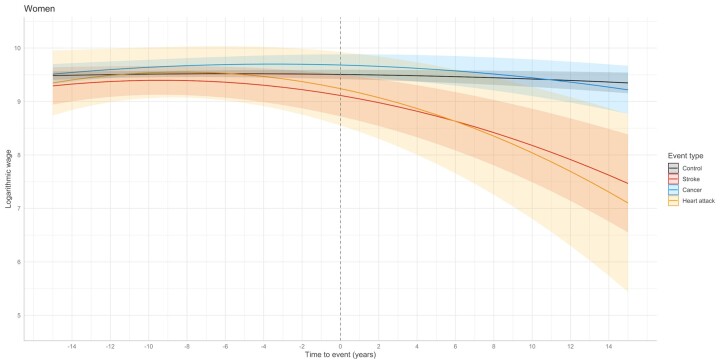
Predicted income development before and after severe health event compared to matched controls in women

At the time of severe health event, women with stroke, heart attack, or cancer had similar income to their matched controls (table 2, figure 1). Getting stroke or heart attack associated with decreasing income trend compared to controls (estimate −0.54, 95% CI −0.88 to −0.20 in stroke group, and −0.68, 95% CI −1.28 to −0.09 in heart attack group). The trend accelerated by time with quadratic effect of −0.27 (95% CI −0.43 to −0.12) in stroke group and −0.40 (95% CI −0.66 to −0.13) in heart attack group. The mean logarithmic incomes were 8%, 13%, and 20% lower in stroke group compared to controls 5, 10, and 15 years after the health event, respectively (Supplementary table S3). In heart attack group, the mean logarithmic incomes were 8%, 15%, and 24% lower. Getting cancer did not associate with decreasing income trend among women.

**Table 2:** Time-to-event mixed models evaluating the sex-specific development of income 15 years before and after severe health event compared to matched controls

		95% confidence interval		
	Point estimate	Lower limit	Upper limit	*P* value
**Women**				
Intercept	9.49	9.37	9.61	<0.001
Severe health event				
Stroke	−0.38	−0.79	0.04	0.073
Heart attack	−0.25	−0.96	0.46	0.486
Cancer	0.19	−0.04	0.43	0.111
Time-to-event				
Controls	−0.07	−0.16	0.02	0.107
Stroke	−0.54	−0.88	−0.20	0.002
Heart attack	−0.68	−1.28	−0.09	0.025
Cancer	−0.03	−0.21	0.15	0.751
Time-to-event^2^				
Controls	−0.06	−0.11	0.00	0.053
Stroke	−0.27	−0.43	−0.12	<0.001
Heart attack	−0.40	−0.66	−0.13	0.003
Cancer	−0.09	−0.17	0.00	0.052
**Men**				
Intercept	9.60	9.46	9.73	<0.001
Severe health event				
Stroke	−1.10	−1.57	−0.62	<0.001
Heart attack	−0.33	−0.80	0.13	0.157
Cancer	−0.03	−0.39	0.34	0.877
Time-to-event				
Controls	−0.24	−0.34	−0.14	<0.001
Stroke	−1.00	−1.37	−0.63	<0.001
Heart attack	−0.69	−1.05	−0.32	<0.001
Cancer	−0.26	−0.55	0.04	0.085
Time-to-event^2^				
Controls	−0.10	−0.18	−0.03	0.015
Stroke	−0.22	−0.39	−0.04	0.014
Heart attack	−0.24	−0.42	−0.07	0.006
Cancer	−0.14	−0.28	0.01	0.061

The superscript number 2 refers to model with a quadratic term of time-to-event.

**Figure 2:**
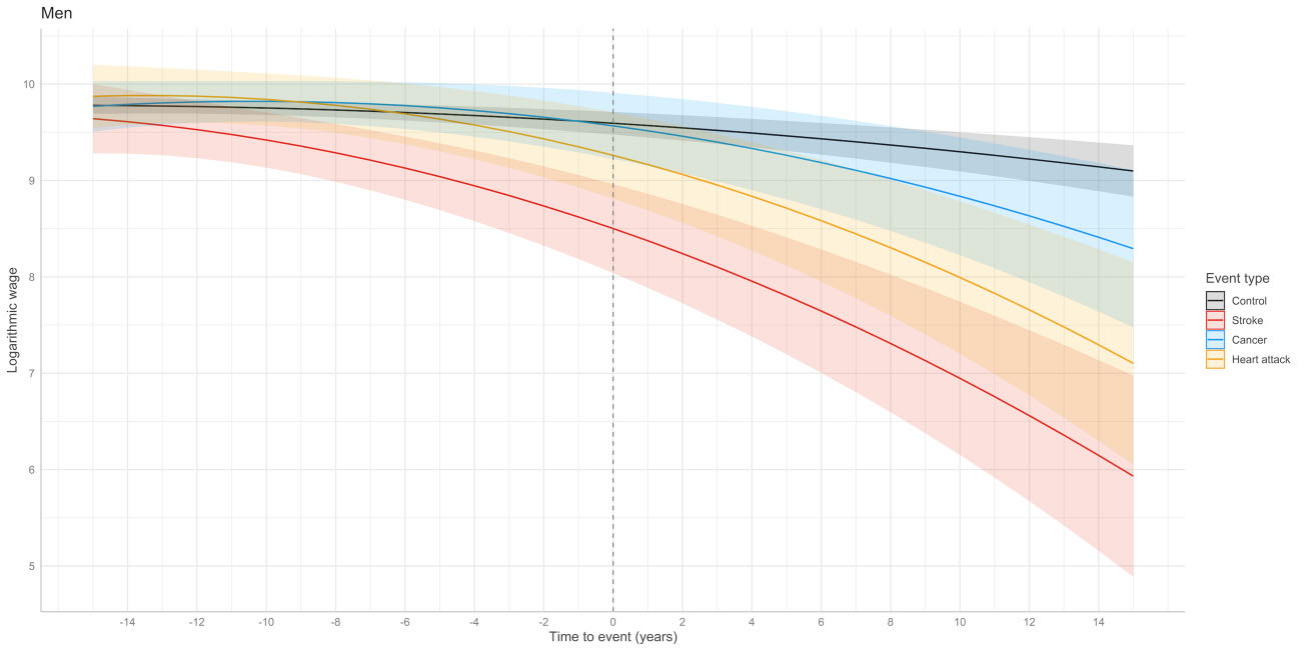
Predicted income development before and after severe health event compared to matched controls in men

Among men, those with stroke had decreasing income trend already at the time of health event (−1.10; 95% CI −1.57 to −0.62) (table 2, figure 2). Men in control group (−0.24, 95% CI −0.34 to −0.14), in stroke group (−1.00, 95% CI −1.37 to −0.63) and in heart attack group (−0.69, 95% CI −1.05 to −0.32) had decreasing income trend. Furthermore, the trend accelerated by time with quadratic effect of −0.10 (95% CI −0.18 to −0.03) in control group, −0.22 (95% CI −0.39 to −0.04) in stroke group, and −0.24 (95% CI −0.42 to −0.07) in heart attack group. Compared to controls, those in stroke group had 18%, 25%, and 35% lower mean logarithmic income 5, 10, and 15 years after the health event, respectively (Supplementary table S3). Heart attack group had 8%, 14%, and 22% lower mean logarithmic income. Cancer did not associate with decreasing income trend.

## Discussion

In this life course study following a birth cohort sample of about 12 000 individuals until midlife, we compared persons caught with severe health events to persons with similar starting points of life to investigate the exogenic impacts of severe health events on persons’ individual income. Both among women and men, stroke and heart attack associated with accumulating negative income trend compared to controls. The negative income trend was seen years after the initial event. Cancer did not associate with decreased income trend.

The finding that stroke and heart attack associate with decreased individual income is in line with previous literature.^3^ In this study, we focused solely on the exogenic income effects of severe health events. Even when the effects of starting points in life are controlled for, stroke and heart attack have negative effect on individual income which persists over years, and even accelerates. Here the income was normalized to logarithmic curve for better statistical adequacy. In contrast to stroke and heart attack, cancer does not seem to be associated with decreased income trend compared to persons with similar starting points of life. The finding is in line with previous literature that working-aged cancer survivors are likely to return back to paid work after their cancer has been treated.^23^ In contrast, return to work after stroke or heart attack is less likely.^24^^,^^25^ Furthermore, a previous study has shown that cancer is less likely to affect its survivors’ earnings than their work status.^26^ In that study, the effects of cancer on earnings were found to be different depending on the cancer type, i.e. annual earnings were lower in cancers associated with low survival rates. Also, the effects of cancer on work status were different depending on the age at cancer onset. In the current study, only severe health events below age 53 years were studied, and the age at onset was similar in all severe health events. Differently from most previous studies that have included only severe health even survivors, our study investigated income effects for all persons who had income in adult age. For persons who did not survive, the income effects were investigated until death. Notably, the mortality rates were similar in all severe health events (data not shown) which suggests that mortality does not explain the difference between cancer and cardiovascular events.

Similar individual income effects of severe health events were found both in women and men. In contrast, previous studies have found that women are more likely to leave employment after a severe health event than men.^3^ In our sample, 18% of women and 23% of men who were working at the time of severe health event did not work one year after the event. Differences in post-event working patterns might be due to social factors, but also due to biological differences in diseases themselves and differences in the onset ages, as e.g. cardiovascular diseases start occurring later in women than in men. In this study, women had higher cancer incidence and lower heart attack incidence than men. Also, e.g. the cancer types and typical age at cancer onset differ between women and men.^27^ It should be noted that the perspective of the study was the individual income development and spouses’ income were beyond the scope. It has been previously reported that severe health event does not affect the employment status or income of a spouse or partner.^28^ Therefore, we believe that our results can be generalized to household income.

We found a negative individual income trend also among men in the control group. The control group was selected from the entire birth cohort population using propensity score matching based on similar starting points in life to those experienced in the severe health event group. The hypothesis was that explored health shocks are not exogenous, i.e. shared risk factors since birth are associated with increased risk for severe health events and with negative income trend. However, during the study period the income trend was found to be negative in the entire male population of Northern Finnish origin. This highlights that together with the severe health events and their risk factors affecting persons’ income, external factors such as societal changes affect the individual income. These societal effects might be linked to economic depression in 2008–09 which corresponds to the mean age of severe health event onset in the sample. For future studies it would be important to address causal link between severe health events and individual income. Noteworthy, the societal or employers’ losses due to reduced productivity were not considered in this study.

It was observed in this study that a large proportion of those who got a severe health event did not leave work altogether. The effects of severe health events on more refined employment patterns, e.g. working hours or occupational mobility, were out of the scope of the current study. Notably, the outcome measure, taxable annual individual income, includes wage and salary earnings, self-employment income, as well as public income transfer payments and social security benefits. We could not distinct the type of income from the register data. Capital income was not included in this study. Likely, cohort members too ill to work full- or part-time after the severe health event received social security benefits to defray the losses in earned income. The social security benefits are in Finland mainly earnings-based and can be considered substantial. For example, the average amount of the earnings-related sickness allowance is about 70% of the employee’s prior earnings.^29^ However, we omitted the individual perspective in this study that considers more the amount of income instead of the manner how the income was gained. Respecting the fact that the unique characteristics of national economies, labour markets, social security and health care systems influence studies alike, we understand that the findings are best generalizable to populations living in European welfare societies.

Our study has some limitations. The numbers of cases in severe health event subtypes, e.g. different cancer types, were small and therefore not studied separately. Furthermore, the number of women with heart attack was small (*n* = 35) and these results should be interpreted with caution. The national-level register data did not include information on the disease severity or recurrence, symptoms, specific location, or given treatments. For future studies it would be important to have more information on the severity of illness and survivors’ ability to function, to explore whether the more severe health events affect income to a larger degree. Lastly, income data were available only from 1995 to 2016.

A strength of this study is the use of a large, unselected, population-based birth cohort of over 12 000 people. Our sample is representative of the general Finnish population. Despite the reduced genetic variation in this population,^30^ stroke, heart attack, and cancer incidences are similar to other western populations. The prospective data collection of the cohort started from the second trimester of antenatal period and the follow-up lasted up to 50 years of age. The information on severe health event diagnoses was collected from comprehensive, prospective nationwide registers based on medical records, and was complete for the entire cohort. The questionnaire and clinical examination data were combined with register data to match persons with severe health events with controls with similar starting points of life. Finally, multiple imputation was used to complete the missing data.

Overall, the findings show that stroke and heart attack have negative effects on individual income regardless of early-life factors. The effects are seen years afterwards and they accelerate by time, highlighting the importance of public health measures to prevent cardiovascular events. Furthermore, negative income trend in control men shows that severe health events do not explain all decrease in income. Measures to decrease health and income inequity should start already in early life.

## Supplementary data

Supplementary data are available at *EURPUB* online.

## Funding

NFBC1966 received financial support from University of Oulu [65354, 24000692]; Oulu University Hospital [2/97, 8/97, 24301140]; Ministry of Health and Social Affairs [23/251/97, 160/97, 190/97]; National Institute for Health and Welfare [54121]; and European Regional Development Fund [539/2010 A31592]. This work was supported by Finnish Work Environment Fund. The funders had no role in study design, data collection and analysis, decision to publish or preparation of the manuscript.

*Conflicts of interest*: None declared.

Key points

- Stroke and heart attack cause accumulating negative income trend irrespective of known risk factors in early life course.
- Cancer does not have effect on individual income.
- Severe health events do not explain all decrease in individual income.
- Interventions are needed to reduce individual income gap after heart attack and stroke for people in prime working age.

## Supplementary Material

ckac110_Supplementary_Data

## References

[1] Lenhart O. The effects of health shocks on labor market outcomes: evidence from UK panel data. Eur J Health Econ 2019;20:83–98. 29876764 10.1007/s10198-018-0985-z PMC6394599

[2] Jones AM , Rice N, Zantomio F. Acute health shocks and labour market outcomes: evidence from the post crash era. Econ Hum Biol 2020;36:100811. 31521566 10.1016/j.ehb.2019.100811

[3] García-Gómez P , van Kippersluis H, O’Donnell O, van Doorslaer E. Long term and spillover effects of health shocks on employment and income. J Hum Resour 2013;48:873–909. 25067853 10.1353/jhr.2013.0031 PMC4110210

[4] Garland A , Jeon SH, Stepner M, et al Effects of cardiovascular and cerebrovascular health events on work and earnings: a population-based retrospective cohort study. Can Med Assoc J 2019;191:E3–10. 30617227 10.1503/cmaj.181238 PMC6312519

[5] García-Gómez P. Institutions, health shocks and labour market outcomes across Europe. J Health Econ 2011;30:200–13. 21167616 10.1016/j.jhealeco.2010.11.003

[6] Lechner M , Vazquez-Alvarez R. The effect of disability on labour market outcomes in Germany. Applied Economics 2011;43:389–412.

[7] Lynch J , Smith GD. A life course approach to chronic disease epidemiology. Annu Rev Public Health 2005;26:1–35. 15760279 10.1146/annurev.publhealth.26.021304.144505

[8] Kivelä M , Rissanen I, Kajantie E, et al Pregnancy risk factors as predictors of offspring cerebrovascular disease: the Northern Finland Birth Cohort Study 1966. Stroke 2021;52:1347–54. STROKEAHA120031618. 33626905 10.1161/STROKEAHA.120.031618

[9] Okasha M , McCarron P, Gunnell D, Davey Smith G. Exposures in childhood, adolescence and early adulthood and breast cancer risk: a systematic review of the literature. Breast Cancer Res Treat 2003;78:223–76. 12725422 10.1023/a:1022988918755

[10] Batty GD , Leon DA. Socio‐economic position and coronary heart disease risk factors in children and young people: evidence from UK epidemiological studies. Eur J Public Health 2002;12:263–72. 12506501 10.1093/eurpub/12.4.263

[11] Galobardes B , Lynch JW, Davey Smith G. Childhood socioeconomic circumstances and cause-specific mortality in adulthood: systematic review and interpretation. Epidemiol Rev 2004;26:7–21. 15234944 10.1093/epirev/mxh008

[12] Johnson RC , Schoeni RF. The influence of early-life events on human capital, health status, and labor market outcomes over the life course. BE J Econ Anal Policy 2011;11:2521. Available at:https://www.degruyter.com/document/doi/10.2202/1935-1682.2521/html (18 October 2021, date last accessed). 10.2202/1935-1682.2521 PMC3569741 23412970

[13] Bharadwaj P , Bietenbeck J, Lundborg P, Rooth DO. Birth weight and vulnerability to a macroeconomic crisis. J Health Econ 2019;66:136–44. 31181455 10.1016/j.jhealeco.2019.05.001

[14] University of Oulu: Northern Finland Birth Cohort 1966. University of Oulu. http://urn.fi/urn:nbn:fi:att:bc1e5408-980e-4a62-b899-43bec3755243.

[15] Nordström T , Miettunen J, Auvinen J, et al Cohort profile: 46 years of follow-up of the Northern Finland Birth Cohort 1966 (NFBC1966). Int J Epidemiol 2021;50:1786–1787. 34999878 10.1093/ije/dyab109 PMC8743124

[16] Smith JP. The impact of childhood health on adult labor market outcomes. Rev Econ Stat 2009;91:478–89. 23585697 10.1162/rest.91.3.478 PMC3625038

[17] The Finnish Tax Administration [Internet]. Available at: https://www.vero.fi/en (16 September 2021, date last accessed).

[18] Suomen virallinen tilasto (SVT): Kuluttajahintaindeksi [Internet]. ISSN=1796-3524. *Rahanarvonkerroin 1860–2019.* Helsinki: Tilastokeskus, 2019. Available at: http://www.stat.fi/til/khi/2019/khi_2019_2020-01-16_tau_001.html (18 October 2021, date last accessed).

[19] Finnish Centre for Pensions [Internet]. Available at: https://www.etk.fi/en/ (16 September 2021, date last accessed).

[20] van Buuren S , Groothuis-Oudshoorn K. mice: multivariate imputation by chained equations in R. J Stat Soft 2011;45:1–67.

[21] Pishgar F , Greifer N, Leyrat C, Stuart E. MatchThem:: matching and weighting after multiple imputation. R J 2021;13:228.

[22] Bates D , Mächler M, Bolker B, Walker S. Fitting linear mixed-effects models using lme4. J Stat Soft 2015;67:1–48.

[23] Stone DS , Ganz PA, Pavlish C, Robbins WA. Young adult cancer survivors and work: a systematic review. J Cancer Surviv 2017;11:765–81. 28478587 10.1007/s11764-017-0614-3 PMC5671547

[24] Edwards JD , Kapoor A, Linkewich E, Swartz RH. Return to work after young stroke: a systematic review. Int J Stroke 2018;13:243–56. 29189108 10.1177/1747493017743059

[25] Sun W , Gholizadeh L, Perry L, et al Factors associated with return to work following myocardial infarction: a systematic review of observational studies. J Clin Nurs 2021;30:323–40. 33179345 10.1111/jocn.15562

[26] Jeon SH. The long-term effects of cancer on employment and earnings: the long-term effects of cancer on employment and earnings. Health Econ 2017;26:671–84. 27045223 10.1002/hec.3342

[27] Kim HI , Lim H, Moon A. Sex differences in cancer: epidemiology, genetics and therapy. Biomol Ther (Seoul) 2018; 26:335–42. 29949843 10.4062/biomolther.2018.103 PMC6029678

[28] Jeon SH , Stepner M, Rotermann M, et al Effects of cardiovascular health shocks on spouses’ work and earnings: a national study. Med Care 2020;58:128–36. 31935200 10.1097/MLR.0000000000001249

[29] Mittag O , Kotkas T, Reese C, et al Intervention policies and social security in case of reduced working capacity in the Netherlands, Finland and Germany: a comparative analysis. Int J Public Health 2018;63:1081–8. 29926126 10.1007/s00038-018-1133-3

[30] Sabatti C , Service SK, Hartikainen AL, et al Genome-wide association analysis of metabolic traits in a birth cohort from a founder population. Nat Genet 2009;41:35–46. 19060910 10.1038/ng.271 PMC2687077

