# Supplementary material for: Lost individual income due to severe health events: life-course perspective in the Northern Finland Birth Cohort 1966
Source: Eur J Public Health. 2022 Aug 30;32(5):723–8. doi: 10.1093/eurpub/ckac110 (PMC9527968; doi:10.1093/eurpub/ckac110)
Supplement: ckac110_Supplementary_Data [file ckac110_supplementary_data.zip › ejph-2022-01-om-0055-File004.docx]

**Supplementary material**

**Supplementary Table 1.** Classification of health shocks according to codes in International Classification of Diseases (ICD) versions 8, 9 and 10.

|  | **ICD-8 codes** | **ICD-9 codes** | **ICD-10 codes** |
| --- | --- | --- | --- |
| Stroke | 430-438 | 430-436 | I60-I64 |
| Heart attack | 410-411 | 410-411 | I21-I24 |
| Cancer | 140-207 | 140-208 | C00-C97 |
| Years in use in Finland | 1967-1986 | 1987-1995 | 1996- |
| ICD = International Classification of Diseases. | | | |

**Supplementary Table 2.** Socioeconomic characteristics of sample.

|  | All | Women (N=5273) | | | | | Men (N=5488) | | | | | |
| --- | --- | --- | --- | --- | --- | --- | --- | --- | --- | --- | --- | --- |
|  | Total sample (N=10761) | Unmatched Control (N=2513) | Control (N=2204) | Stroke (N=108) | Cancer (N=413) | Heart attack (N=35) | | Unmatched Control (N=3296) | Control (N=1753) | Stroke (N=113) | Cancer (N=206) | Heart attack (N=120) |
| **Income in 1995** | | | | | | | | | | | | |
| Mean (SD) | 18598 (10768) | 17230 (9368) | 16178.5 (8276) | 15059 (7139) | 16311 (8733) | 15208 (7716) | | 20795 (12346) | 20053 (11350) | 18417 (14222) | 20166 (11810) | 19680 (11717) |
| Median (Q1, Q3) | 17470 (11127, 24274) | 16330 (10906, 22342) | 15355 (10354, 20812) | 15022 (10522, 19941) | 15324 (10351, 21703) | 13699 (11279, 18957) | | 20165 (12279, 27445) | 19006 (12003, 26979) | 17201 (9264, 25105) | 18870 (11589, 26063) | 17773 (12250, 25525) |
| **Income in 2005** | | | | | | | | | | | | |
| Mean (SD) | 29023 (22618) | 25407 (16326) | 23512 (13413) | 21080 (11497) | 25061 (15316) | 20930 (10847) | | 34252 (30464) | 32690 (22005) | 26954 (17284) | 31348 (18786) | 31073 (27119) |
| Median (Q1, Q3) | 26436 (16262, 36353) | 24219 (14939, 31658) | 23054 (14029, 29879) | 20351 (13594, 26229) | 24480 (14579, 31175) | 20768 (12494, 30121) | | 30578 (20008, 42577) | 29720 (18633, 41437) | 27484 (10907, 37053) | 29495 (18163, 41088) | 26919 (17132, 37230) |
| **Income in 2016** | | | | | | | | | | | | |
| Mean (SD) | 33798 (27509) | 31289 (21809) | 29246 (19688) | 19055 (13345) | 30508 (23333) | 22178 (13906) | | 38377 (34592) | 37116 (28608) | 25474 (20552) | 36911 (26418) | 28319 (29518) |
| Median (Q1, Q3) | 30326 (19471, 42404) | 29291 (20074, 38793) | 27841 (18820, 36299) | 19400 (6932, 28269) | 28705 (19293, 37321) | 24890 (10436, 31779) | | 33215 (20574, 47919) | 33590 (20182, 47307) | 23584 (9169, 39856) | 32730 (19598, 47761) | 26041 (11369, 36421) |
| **At least third-degree education** | | | | | | | | | | | | |
| N (%) | 4665 (43.4) | 1405 (55.9) | 1079 (49.0) | 46 (42.6) | 209 (50.6) | 9 (25.7) | | 1229 (37.3) | 571 (32.6) | 30 (26.5) | 63 (30.6) | 24 (20.0) |
| **Age at time of health shock** | | | | | | | | | | | | |
| Mean (SD) | 43.0 (8.1) | - | - | 43.5 (8.1) | 42.0 (8.2) | 44.5 (8.3) | | - | - | 42.5 (8.2) | 43.7 (8.3) | 44.8 (6.3) |
| Median (Q1, Q3) | 45.0 (38.0, 49.5) | - | - | 46.0 (40.0, 49.0) | 44.0 (36.0, 49.0) | 47.0 (43.5, 50.0) | | - | - | 45.0 (38.0, 49.0) | 46.0 (40.0, 50.0) | 46.0 (42.0, 50.0) |
| **Socioeconomic status at the time of severe health event, n (%)** | | | | | | | | | | | | |
| Farmers | 265 (2.6) | 54 (2.2) | 26 (1.3) | 2 (2.0) | 7 (1.8) | 2 (6.5) | | 107 (3.3) | 57 (3.5) | 2 (1.9) | 4 (2.1) | 4 (3.5) |
| Self-employed persons | 702 (6.8) | 117 (4.7) | 106 (5.3) | 6 (6.1) | 24 (6.3) | 0 (0.0) | | 269 (8.2) | 136 (8.4) | 11 (10.6) | 16 (8.5) | 17 (14.8) |
| Upper-level white collar employees | 2089 (20.2) | 568 (22.8) | 360 (17.8) | 7 (7.1) | 73 (19.1) | 3 (9.7) | | 727 (22.2) | 288 (17.8) | 14 (13.5) | 37 (19.6) | 12 (10.4) |
| Lower-level white-collar employees | 2975 (28.8) | 1053 (42.3) | 810 (40.1) | 44 (44.9) | 154 (40.3) | 13 (41.9) | | 577 (17.6) | 276 (17.0) | 13 (12.5) | 21 (11.1) | 14 (12.2) |
| Manual workers | 2285 (22.1) | 325 (13.0) | 289 (14.3) | 9 (9.2) | 51 (13.4) | 3 (9.7) | | 987 (30.1) | 497 (30.7) | 23 (22.1) | 61 (32.3) | 40 (34.8) |
| Students | 241 (2.3) | 62 (2.5) | 70 (3.5) | 4 (4.1) | 15 (3.9) | 0 (0.0) | | 53 (1.6) | 25 (1.5) | 3 (2.9) | 7 (3.7) | 2 (1.7) |
| Pensioners | 526 (5.1) | 96 (3.9) | 93 (4.6) | 12 (12.2) | 18 (4.7) | 5 (16.1) | | 169 (5.2) | 99 (6.1) | 13 (12.5) | 12 (6.3) | 9 (7.8) |
| Others | 983 (9.5) | 151 (6.1) | 208 (10.3) | 12 (12.2) | 29 (7.6) | 2 (6.5) | | 321 (9.8) | 201 (12.4) | 19 (18.3) | 26 (13.8) | 14 (12.2) |
| Unknown | 695 (6.5) | 87 (3.5) | 242 (11.0) | 12 (11.1) | 42 (10.2) | 7 (20.0) | | 86 (2.6) | 174 (9.9) | 15 (13.3) | 22 (10.7) | 8 (6.7) |
| **Earning days after severe health event, percentage** | | | | | | | | | | | | |
| Mean (SD) | 0.8 (0.3) | 0.8 (0.3) | 0.8 (0.3) | 0.6 (0.4) | 0.7 (0.4) | 0.6 (0.4) | | 0.8 (0.4) | 0.8 (0.4) | 0.6 (0.4) | 0.5 (0.4) | 0.7 (0.4) |
| Median (Q1, Q3) | 1.0 (0.7, 1.0) | 1.0 (0.8, 1.0) | 1.0 (0.7, 1.0) | 0.8 (0.1, 1.0) | 0.9 (0.5, 1.0) | 0.9 (0.1, 1.0) | | 1.0 (0.7, 1.0) | 1.0 (0.6, 1.0) | 0.6 (0.0, 1.0) | 0.7 (0.0, 1.0) | 0.9 (0.2, 1.0) |

SD = standard deviation. Income discounted to year 2019. The socioeconomic status in the year 2008 was selected for unmatched controls. Earning days were calculated after the year 2010 for unmatched controls.

**Supplementary Table 3**. Logarithmic mean annual incomes at the time of severe health event, and 5, 10, and 15 years afterwards.

|  | Time to event (years) | | | |
| --- | --- | --- | --- | --- |
|  | 0 | 5 | 10 | 15 |
| **Women** | | | | |
| **Control** |  |  |  |  |
| Log(income) | 9.5 | 9.5 | 9.4 | 9.3 |
| **Stroke** |  |  |  |  |
| Log(income) | 9.1 | 8.7 | 8.2 | 7.5 |
| % difference | -4.1 | -7.9 | -13.2 | -20.1 |
| **Heart attack** |  |  |  |  |
| Log(income) | 9.2 | 8.8 | 8.0 | 7.1 |
| % difference | -2.8 | -7.6 | -14.6 | -24.0 |
| **Cancer** |  |  |  |  |
| Log(income) | 9.7 | 9.6 | 9.4 | 9.2 |
| % difference | +1.9 | +1.3 | +0.2 | -1.4 |
| **Men** | | | | |
| **Control** |  |  |  |  |
| Log(income) | 9.6 | 9.5 | 9.3 | 9.1 |
| **Stroke** |  |  |  |  |
| Log(income) | 8.5 | 7.8 | 6.9 | 5.9 |
| % difference | -11.4 | -17.5 | -25.3 | -34.8 |
| **Heart attack** |  |  |  |  |
| Log(income) | 9.3 | 8.7 | 8.0 | 7.1 |
| % difference | -3.5 | -7.9 | -14.0 | -21.9 |
| **Cancer** |  |  |  |  |
| Log(income) | 9.6 | 9.2 | 8.8 | 8.3 |
| % difference | -0.3 | -2.1 | -5.0 | -8.9 |

Log = logarithm; % difference = percentual difference to control group.

**Supplementary Figure 1.** Causal path diagram of the study concept. To study the association between severe health events and income, we adjusted for potential confounders occurring early in the life-course and stratified the analyses for sex. Possible (latent) mediating factors, such as disability and work absenteeism and presenteeism were not adjusted for.

**Supplementary Figure 2.** Covariate balance of propensity score matching.

**Supplementary Figure 3.** Number of severe health events per year.

**Supplementary Figure 4.** Income development of entire sample.
